# Activation of the mitogen‐activated protein kinase ERK1/2 signaling pathway suppresses the expression of ChREBPα and β in HepG2 cells

**DOI:** 10.1002/2211-5463.13208

**Published:** 2021-06-17

**Authors:** Lan Li, Haruhiko Sakiyama, Hironobu Eguchi, Daisaku Yoshihara, Noriko Fujiwara, Keiichiro Suzuki

**Affiliations:** ^1^ Department of Biochemistry Hyogo College of Medicine Nishinomiya Japan

**Keywords:** ChREBPα, ChREBPβ, ERK, oxidative stress, staurosporine

## Abstract

The carbohydrate response element‐binding protein (ChREBP), a glucose‐responsive transcription factor that plays a critical role in the glucose‐mediated induction of genes involved in hepatic glycolysis and lipogenesis, exists as two isoforms: ChREBPα and ChREBPβ. However, the mechanism responsible for regulating the expression of both ChREBPα and β, as well as the mechanism that determines which specific isoform is more responsive to different stimuli, remains unclear. To address this issue, we compared the effects of several stimuli, including oxidative stress, on the mRNA and protein expression levels of ChREBPα and β in the hepatocyte cell line, HepG2. We found that H_2_O_2_ stimulation suppressed the expression of both mRNA and protein in HepG2 cells, but the mRNA expression level of ChREBPβ was < 1% of that for ChREBPα levels. In addition, the reduction in both ChREBPα and β mRNA levels was reversed by PD98059, a selective and cell permeable inhibitor of the MEK/ERK pathway. Additionally, the administration of 12‐O‐tetradecanoylphorbol 13‐acetate (TPA) and staurosporine (STS), activators of extracellular‐signal‐regulated kinase (ERK) signaling, also resulted in a decrease in the levels of both ChREBPα and β mRNA in HepG2 cells through ERK signaling. These collective data suggest that oxidative stress, including STS treatment, suppresses the expression of ChREBPα and β via the activation of ERK signaling in HepG2 cells. Such a decrease in the levels of expression of ChREBPα and β could result in the suppression of hepatic glycolysis and lipogenesis, and this would be expected to prevent further oxidative stress.

## Graphical abstract

The carbohydrate response element binding protein (ChREBP) is a glucose‐responsive transcription factor that strongly regulates glycolysis and lipogenesis. In this study, we showed that oxidative stress, including STS and TPA treatment, suppressed the expression of both ChREBPα and β through activation of ERK signaling in HepG2 cells.

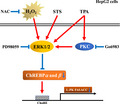

**Abbreviations**
ACC: acetyl‐CoA carboxylase
AGEs: advanced glycation end products
ChREBP: carbohydrate response element‐binding protein
ERK: extracellular‐signal‐regulated kinase
FAS: fatty acid synthase
GK: glucokinase
L‐PK: Liver‐type pyruvate kinase
MAPK: mitogen‐activated protein kinase
Mlx: max‐like protein
NAC: N‐acetyl‐l‐cysteine
O‐GlcNAc: O‐linked N‐acetylglucosamine
PFK: phosphofructokinase
PP2A/PKA: protein phosphatase 2A/protein kinase A
ROS: reactive oxygen species
SREBP: sterol regulatory element‐binding proteins
STS: staurosporine
TPA: 12‐O‐tetradecanoylphorbol 13‐acetate
TXNIP: thioredoxin‐interacting protein

Reactive species, which mainly include reactive oxygen species (ROS), are products generated in mitochondria and in glucose metabolism in eukaryotic cells [1]. Excessive ROS levels are thought to be the major cause of macromolecular damage and have been implicated in the development of numerous diseases. ROS production is strongly dependent on the mitochondrial respiratory chain, a system that is greatly influenced by upstream glycolysis [2, 3]. Glycolysis, in turn, is also known to be significantly involved in oxidative stress by virtue of its ability to change the ratio of NAD^+^ to NADH [4]. Glycolysis is largely regulated by three enzymes, glucokinase (GK), phosphofructokinase (PFK), and liver‐type pyruvate kinase (L‐PK), which function as rate‐limiting enzymes [5]. The L‐PK promoter is positively regulated by the carbohydrate response element‐binding protein (ChREBP), a member of the basic helix‐loop‐helix family of transcription factors and transactivating glucose‐responsive genes by binding to DNA as a heterodimer with the Max‐like protein (Mlx) at a well‐defined carbohydrate‐responsive element. This transcription factor has recently been reported to be a key determinant in controlling hepatic glycolysis and de novo fatty acid synthesis in the liver under physiological conditions [6].

The activation of ChREBP through nuclear/cytosol shuttling in response to metabolites derived from glucose is a subject that is an object of considerable interest. Briefly, inactive ChREBP is dephosphorylated by Xu‐5‐P/PP2A and is then translocated into the nucleus through interaction with importinα, thus inducing the transcriptional expression of L‐PK and lipogenic genes. In addition, starving signals such as the glucagon driven nuclear export signal are dependent on PKA phosphorylation and complex formation with 14‐3‐3 [7, 8, 9]. In addition, our group previously reported that O‐linked N‐acetylglucosamine (O‐GlcNAc) modification also increased the glucose response of ChREBP [10]. In contrast, few studies regarding the transcriptional regulation of ChREBP have appeared. It was reported that the liver x receptor (LXR)/ retinoid x receptor (RXR) complex and the thyroid hormone receptor (TR) binds to the promoter of ChREBP and induces its expression in the liver and white adipose tissue (WAT) [11, 12]. Aside from its role in the regulation of metabolism, recent studies have also shed light on nonmetabolic factors that trigger the transcriptional regulation of ChREBP. For example, ChREBP has been shown to mediate the glucose induced expression of the thioredoxin‐interacting protein (TXNIP), which induces oxidative stress in pancreatic β cells [13]; and that the mTOR‐regulated ChREBP/TXNIP pathway plays an important role in inhibiting diabetes stress in β cells for cell survival [14].

More recently, reports have emerged showing that a distinct cellular response to nutrients exists between ChREBPα and β, two isoforms of ChREBP that are transcribed from different promoters. In mouse adipose tissue, the glucose‐mediated activation of ChREBPα induces the expression of ChREBPβ isoform, which is located exclusively in the nucleus. The transcriptional activity of ChREBPβ is 20‐fold greater than that of ChREBPα at high glucose concentrations, indicating a feed‐forward mechanism and that ChREBPβ may be a more potent activator of ChREBP transcriptional target genes [15]. When mice were subjected to the fasting–refeeding of high carbohydrate diets, ChREBPα was not induced, even though ChREBPβ levels were increased by 10‐ to 20‐fold [16]. In addition, Meng *et al*. [17] reported that HNF‐4α promoted the transcription of ChREBP in response to glucose and HNF‐4α and ChREBPα coordinately increased the transcription of ChREBPβ. Whereas in diabetic mice, islet ChREBPβ was increased, which downregulates ChREBPα via a negative feedback loop to ultimately limit the excessive glucose induced by the ChREBPα‐mediated gene expression [18]. In another study, Chen *et al*. [19] reported that advanced glycation end products (AGEs) promoted the expression of both ChREBPα and β through increasing ROS levels in HepG2 cells, suggesting that oxidative stress may regulate the expression of ChREBP, but the mechanism responsible for this remains unknown. From the above, it would appear that the responsiveness of ChREBP varies depending on the tissue and the type of stimulus. Furthermore, in terms of their contributions for ChREBP‐attributed metabolic functions, it will be crucial to define specific regulators of ChREBPα and β.

Studies have shown that oxidative stress can cause organ‐specific and path‐specific toxicity, and plays a key role in cell proliferation, tumor development, and glucose/lipid metabolism [20, 21, 22, 23]. Furthermore, the exposure of cells to exogenous H_2_O_2_ mimics oxidative stress and leads to the activation of the mitogen‐activated protein kinase (MAPK) [24]. The MAPK pathways consist of a family of protein kinases including extracellular‐signal‐regulated kinase (ERKs), c‐jun N‐terminal kinases (JNKs), and p38, which play a crucial role in signaling transduction from the cell surface to the nucleus. The ERK cascade is induced by a variety of extracellular agents including growth factors, and hormones as well as oxidative stress. ERK activation following H_2_O_2_ stimulation has been reported to be involved in cell survival following oxidative injury in several cell lines, and these effects can be abolished by ERK inhibitors and antioxidants N‐acetyl‐l‐cysteine (NAC) [24, 25, 26].

This study focused primarily on HepG2 cells and investigated the effects of noncarbohydrate stimulation, especially oxidative stress, on the expression levels of ChREBPα and β. Based on our findings, we report on a novel mechanism by which the activation of the ERK signaling cascade suppresses the transcriptional expression of both ChREBP isoforms in hepatocytes.

## Materials and methods

### Chemicals

All chemicals used in this study were purchased from either Wako Pure Chemical Industries Ltd. (Osaka, Japan), Nacalai Tesque, Inc. (Kyoto, Japan), Abcam Ltd. (Cambridge, UK), or Sigma‐Aldrich, unless specified otherwise, and were of the highest grade available. Go6983 (PKC inhibitor) was purchased from Abcam (Cambridge, UK). 12‐O‐tetradecanoylphorbol 13‐acetate (TPA) (PKC activator) and PD98059 (MEK inhibitor) were obtained from Cell Signaling Technology (Beverly, MA, USA). Staurosporine (STS) (a non‐specific protein kinase inhibitor) was purchased from Cayman Chemical (Ann Arbor, MI, USA). Thirty % H_2_O_2_ was purchased from Nacalai Tesque (Kyoto, Japan). NAC was obtained from WAKO (Osaka, Japan). STS was dissolved in ethyl acetate (WAKO, Osaka, Japan), and other compounds, unless otherwise specified, were dissolved in DMSO (WAKO, Osaka, Japan) and added directly to the culture medium.

### Cell culture and treatment

Human hepatocyte carcinoma cell line HepG2 cells were maintained in Dulbecco's modified Eagle's medium (DMEM; Invitrogen, Carlsbad, CA, USA) supplemented with 5% fetal bovine serum, 100 U·mL^−1^ penicillin, and 100mg/ml streptomycin at 37 °C under an atmosphere of 95% air and 5% CO_2_. The cells were plated in 6‐well plates at a density of 2 x 10*5·mL^−1^ the day before treatment. Unless otherwise specified, the cells were pretreated with the indicated concentrations of Go6983 or PD98059 or NAC for 1 h, and then stimulated by H_2_O_2_/STS/TPA for an additional 24 h.

### Western blotting analysis

HepG2 cells were washed with phosphate‐buffered saline (PBS) twice and lysed by treatment with RIPA buffer (Fujifilm Pure Chemical Industries Ltd., Osaka, Japan). Cell lysates were centrifuged at 4 °C and 20 000 x ***g*** for 20 min. Supernatants were collected and quantified by means of a Pierce ^TM^ BCA Protein Assay Kit (Thermo scientific USA Ltd., Rockford, IL, USA). Protein extracts were size‐fractionated on a 5%–20% SDS/PAGE gel and transferred to a polyvinylidene fluoride difluoride (PVDF) membrane (GE Healthcare UK Ltd., Buckinghamshire, UK) in a Trans‐Blot® SD Semi‐Dry Electrophoretic Transfer Cell (Bio‐Rad USA Ltd., Redmond, WA, USA). The membrane was then incubated with the primary antibody (phospho‐p44/42 MAPK (ERK1/2), anti‐p44/42 MAPK (ERK1/2), anti‐ β ‐tubulin, and anti‐β‐actin (Cell Signaling Technology, Danvers, USA), 1 : 500; anti‐PK (Gene Tex, Irvine, CA, USA), 1:500; anti‐ChREBP (Novus Biologicals, NB400‐135, USA), 1 : 500) and a secondary antibody Horseradish peroxidase‐conjugated anti‐rabbit IgG (Cell Signaling Technology, USA 1 : 500) overnight in an iBind Western System (Thermo scientific, Rockford, IL, USA), and proteins were visualized using an ECL Western Blotting Detection System (GE Healthcare, Buckinghamshire, UK). Coomassie Brilliant Blue (quick CBB‐plus, Wako, Osaka, Japan) staining was used as a loading control according to the manufacturer's instructions.

### RNA extraction and reverse transcription

Total RNA was isolated from HepG2 cells using Sepazol RNA‐I super G reagent (Nacalai Tesque, Kyoto, Japan). Total RNA (1 μg) was reverse transcribed using a high‐capacity RNA‐to‐cDNA kit (Applied Biosystems, Foster City, CA, USA) according to the manufacturer's instructions.

### Real‐time PCR

Real‐time PCR was performed with the PowerTrack™ SYBR GreenMaster Mix (Thermo Fisher Scientific) and a Quant Studio 12K Flex Real Time PCR System (Applied Biosystems) with gene‐specific primers or subjected to 2% agarose gel and then stained with ethidium bromide to detect the levels of mRNAs. The sequences of q‐PCR primers (for human sapiens) are listed below.

**Table:** 

ChREBPα	Forward	(5′‐ACTCGGACTCGGACACAGAC‐3′)	Reverse	(5′‐AGGCTCAAGCACTCGAAGAG‐3′)
ChREBPβ	Forward	(5′‐AGCGGATTCCAGGTGAGG‐3′)	Reverse	(5′‐TTGTTCAGGCGGATCTTGTC‐3′)
β‐actin	Forward	(5′‐GTGGCATCCACGAAACTACCTT ‐3′)	Reverse	(5′‐GGACTCGTCATACTCCTGCTTG ‐3′)
FAS	Forward	(5′‐AACCGGCTCTCCTTCTTCTTCGACTT‐3′)	Reverse	(5′‐TCCGAGCGGCAGTACCCATTC‐3′)
ACC	Forward	(5′‐GCAGAATTTGTTACTCGCTTTGG‐3′)	Reverse	(5′‐ATTTCATAAGACCACCTACGGATAGAC‐3′)
L‐PK	Forward	(5′‐AATCCCAGGTTTGGCTGGTT‐3′)	Reverse	(5′‐AGCACGAGTTGTGAAGACAGT‐3′)
PFK	Forward	(5′‐CCACCGTGGACTGTGTCTGTT‐3′)	Reverse	(5′‐GGGCACTCGCCGATTAGGTAT‐3′)
GK	Forward	(5′‐CAACTGGACCAAGGGCTTCAA‐3′)	Reverse	(5′‐CGTGGCCACCGTGTCATTC‐3′)

### Data presentation and statistical methods

Data are expressed as the mean ± SE from a single experiment representative of more than three independent experiments. Statistical analyses were performed with GraphPad Prism 6 using a Student's *t* test or one‐way ANOVA. In all analyses, a *P* value of *P* < 0.05 was considered significant.

## Results and Discussion

### H_2_O_2_ treatment downregulates the gene expression of ChREBPα and β in HepG2 cells

**Fig. 1:**
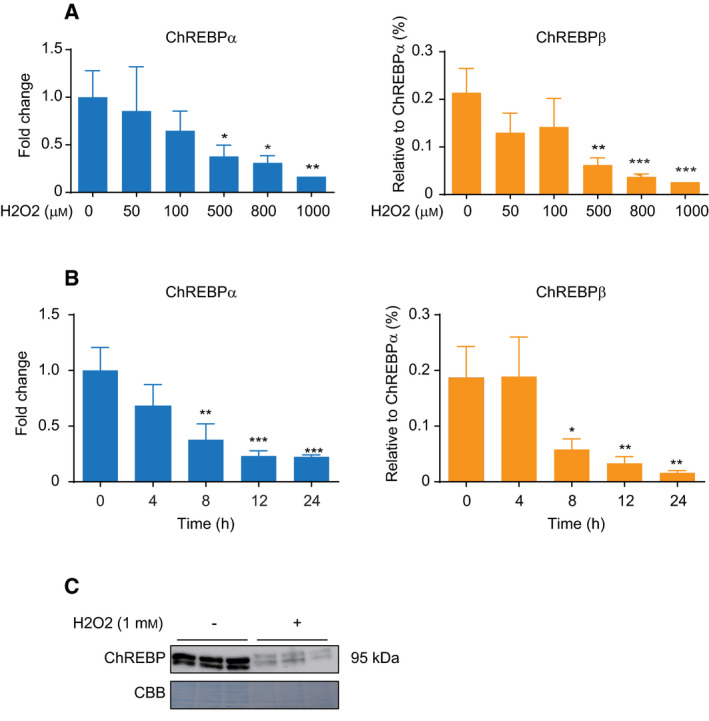
Effect of ROS stress on ChREBPα and β expression. (A) HepG2 cells were exposed to indicated concentrations of H_2_O_2_ for 24 h. The mRNA levels of ChREBPα and β were measured by quantitative RT‐PCR, respectively. Left panel shows the relative expression level of ChREBPα after normalization to β‐actin. The right panel shows the relative expression level of ChREBPβ normalized by ChREBPα. (B) HepG2 cells were incubated with 1 mm H_2_O_2_ for indicated hours. The mRNA levels of ChREBPα and β were measured by quantitative RT‐PCR. Left panel shows the relative expression level of ChREBPα after normalization to β‐actin. The right panel shows the relative expression level of ChREBPβ normalized by ChREBPα. Data from one representative experiment of three independent experiments are presented as the mean ± SE of three technical replicates. (A–B) Student *t* test: *P < 0.05; **P < 0.01; ***P < 0.001 versus normal group. (C) HepG2 cells were treated with or without 1 mm H_2_O_2_ for 24 h. The expression levels of ChREBP were detected by western blotting. A representative Coomassie Blue‐stained blot is shown as a loading control.

To determine the effect of oxidative stress on the gene expression of ChREBP, HepG2 cells were challenged with 1 mm of H_2_O_2_ for 24 h and the mRNA levels of ChREBPα and β were analyzed by comparative analysis of agarose gel electrophoresis. As shown in Fig. S1, we observed that the mRNA levels of ChREBPα were much higher than those of ChREBPβ and that the stimulation with 1 mm H_2_O_2_ reduced both the ChREBPα and β mRNA expressions. The real‐time PCR analysis clearly showed that the addition of H_2_O_2_ suppressed the mRNA levels of both ChREBPα (Fig. 1A, left) and β (Fig. 1A, right) in a dose‐dependent manner and that the mRNA levels of ChREBPβ were < 1% of the former in HepG2 cells (Fig. 1A, right). We therefore conclude that ChREBPα is the major isoform in HepG2 cells. In addition, stimulation with 1 mm H_2_O_2_ reduced the ChREBPα and β mRNA expression in a time‐dependent manner (Fig. 1B). Further analysis by western blotting also showed that the protein expression level of ChREBP was decreased as the result of the H_2_O_2_ treatment (Fig. 1C). These results demonstrate that oxidative stress efficiently reduces the expression of both ChREBPα and β. Since the expression level of ChREBPβ was quite low in the liver, the effect was expected to be extremely small compared to that of ChREBPα. Thus, ChREBPα appears to be a more potent mediator of hepatic ChREBP functions.

### ERK activation is required in H_2_O_2_ suppressed expression of ChREBP in HepG2 cells

**Fig. 2:**
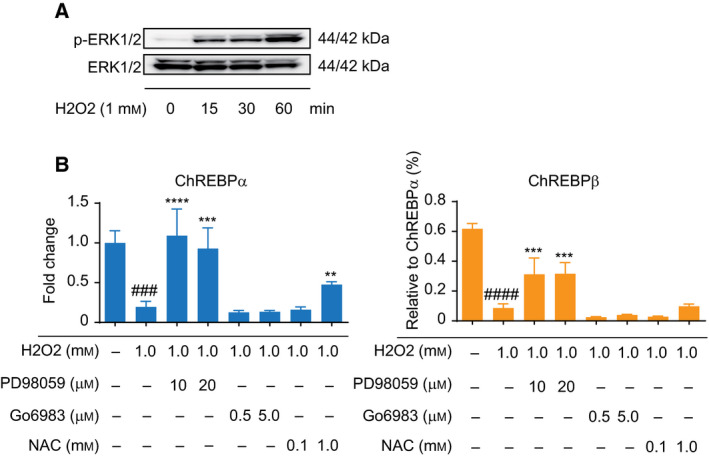
Activation of p‐ERK induced by H_2_O_2_ and the recovery of ChREBPα and β expression by an inhibitor for ERK and NAC, an antioxidant. (A) HepG2 cells were exposed to 1 mm H_2_O_2_ for indicated minutes. The expression levels of ERK and p‐ERK were detected by western blotting. (B) HepG2 cells were pre‐incubated in the presence or absence of PD98059, Go6983 or NAC. After 1 h, cells were treated with 1 mm H_2_O_2_ and incubated for additional 24 h. The mRNA levels of ChREBPα and β were measured by quantitative RT‐PCR, respectively. Left panel shows the relative expression level of ChREBPα after normalization to β‐actin. The right panel shows the relative expression level of ChREBPβ normalized by ChREBPα. Data from one representative experiment of three independent experiments are presented as the mean ± SE of three technical replicates. One‐way ANOVA test: ###*P* < 0.001; ####*P* < 0.0001 versus normal group. **P < 0.01; ***P < 0.001; ****P < 0.0001 versus the H_2_O_2_ group.

To gain a deeper insight into the effects of the H_2_O_2_ treatment on the regulation of ChREBP, the involvement of ERK signaling was evaluated. When HepG2 cells were exposed to 1 mm H_2_O_2_ for the indicated number of minutes, a dramatic activation of ERK was observed after the cells had been exposed to H_2_O_2_ for 15 min and this activation increased with time as detected by western blotting (Fig. 2A). Pretreatment with PD98059, but not Go6983, specific inhibitors of the MAPK (MEK) and PKC, respectively, abolished the effect of H_2_O_2_ stimulation on the expression of both ChREBPα and β, suggesting that PKC‐independent ERK activation was responsible for the reduced expression of ChREBPα and β in response to exposure to H_2_O_2_ (Fig. 2B). On the other hand, the addition of the antioxidant NAC restored the expression of ChREBPα but not that of β caused by the H_2_O_2_ treatment (Fig. 2B). Therefore, these results indicate that ROS including H_2_O_2_ negatively regulates the expression of both ChREBPα and β. On the contrary, Chen *et al*. [19] recently proposed an up‐regulation of ChREBPα and β promoted by AGEs as the result of increasing ROS levels in HepG2 cells. However, it is unclear whether ROS resulting from AGEs activates the expression of ChREBPα and β because glucose increases the expressions of ChREBPα and β, and AGEs contain glucose‐like moieties. In light of these data, ChREBP is at least a potential candidate gene that is affected in response to oxidative stress, and our results provide direct evidence to show that ChREBPα and β is suppressed by oxidative stress in liver cells. Some studies have reported that the activation of ERK by H_2_O_2_ plays a critical role in cell survival following oxidative damage [24, 26] and other studies have reported that ERK activation mediated by ROS induces apoptosis in a variety of cells [27, 28]. Therefore, further study will be required to analyze the markers that are involved in cell survival or cell death regarding ChREBP following H_2_O_2_ exposure.

### TPA treatment downregulates mRNA level of ChREBP through ERK activation in HepG2 cells

**Fig. 3:**
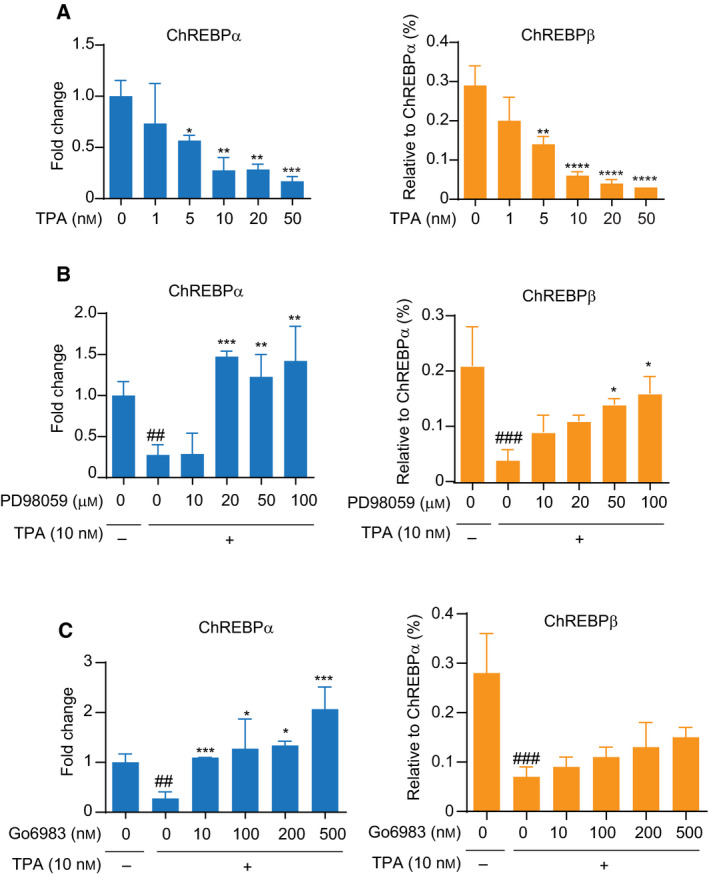
Suppression of mRNA expression of ChREBPα and β by treatment with TPA in HepG2 cells. (A) HepG2 cells were treated with indicated concentrations of TPA for 24 h. The mRNA levels of ChREBPα and β were measured by quantitative real‐time PCR, respectively. Left panel shows the relative expression level of ChREBPα after normalization to β‐actin. The right panel shows the relative expression level of ChREBPβ normalized by ChREBPα. Data from one representative experiment of three independent experiments are presented as the mean ± SE of three technical replicates. Student *t* test: *P < 0.05; **P < 0.01; ***P < 0.001; ****P < 0.0001 versus normal group. (B and C) HepG2 cells were pretreated with PD98059 and Go6983 at the indicated concentrations followed by treatment of 10 nm TPA for 24 h. The mRNA levels of ChREBP were measured by quantitative real‐time PCR. Left panel shows the relative expression level of ChREBPα after normalization to β‐actin. The right panel shows the relative expression level of ChREBPβ normalized by ChREBPα. Data from one representative experiment of three independent experiments are presented as the mean ± SE of three technical replicates. One‐way ANOVA test: ##*P* < 0.01; ###*P* < 0.001 versus normal group. *P < 0.05; **P < 0.01; ***P < 0.001 versus TPA group.

To ascertain the downregulation of ChREBP by ERK activation, we used TPA, which induces the PKC‐dependent activation of ERK in HepG2 cells [29]. Similar to the effect of H_2_O_2_ exposure, the TPA treatment also reduced the mRNA levels of ChREBPα and β in a dose‐dependent manner in HepG2 cells (Fig. 3A). In addition, both a MEK inhibitor (PD98059) and a PKC inhibitor (Go6983) completely restored ChREBPα expression levels and, at high concentrations, increased their expression somewhat (Fig. 3B left and C left). In contrast, ChREBPβ was only partially restored by the ERK inhibitors (Fig. 3B right and C right) although the expression levels were measurably less than ChREBPα. These results demonstrate that PKC‐dependent ERK activation suppresses the expression of ChREBPα.

### STS suppresses expression of ChREBP through ERK activation in HepG2 cells

**Fig. 4:**
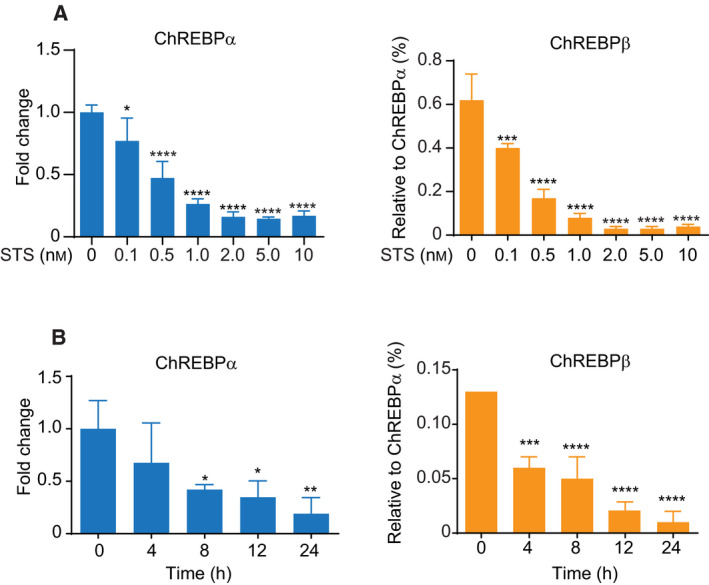
Suppression of mRNA of ChREBPα and β by treatment with STS in HepG2 cells. (A) HepG2 cells were treated with indicated concentrations of STS for 24 h. The mRNA level of ChREBPα and β were measured by quantitative RT‐PCR. Left panel shows the relative expression level of ChREBPα after normalization to β‐actin. The right panel shows the relative expression level of ChREBPβ normalized by ChREBPα. (B) HepG2 cells were challenged with 10 nm STS for indicated hours. The mRNA levels of ChREBPα and β were measured by quantitative RT‐PCR. Left panel shows the relative expression level of ChREBPα after normalization to β‐actin. The right panel shows the relative expression level of ChREBPβ normalized by ChREBPα. Data from one representative experiment of three independent experiments are presented as the mean ± SE of three technical replicates. (A–B) Student *t* test: *P < 0.05; **P < 0.01; ***P < 0.001; ****P < 0.0001 versus normal group.

**Fig. 5:**
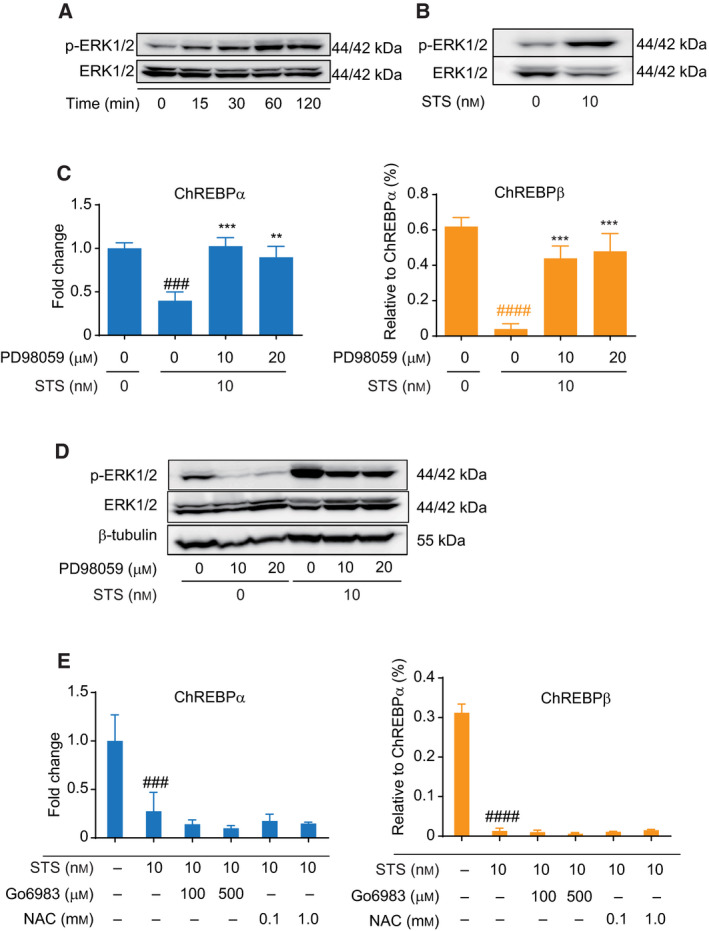
ERK activation is required for the suppression of mRNA of ChREBPα and β by treatment with STS. (A and B) HepG2 cells were treated with 10 nm STS for 0–2 or 24 h. The expression levels of ERK and p‐ERK were detected by western blotting. (C) HepG2 cells were pretreated with indicated concentrations of PD98059 and incubated additional 24 h with or without 10 nm STS. The mRNA levels of ChREBPα and β were measured by quantitative RT‐PCR. Left panel shows the relative expression level of ChREBPα after normalization to β‐actin. The right panel shows the relative expression level of ChREBPβ normalized by ChREBPα. Data from one representative experiment of three independent experiments are presented as the mean ± SE of three technical replicates. One‐way ANOVA test: ###*P* < 0.001; ####*P* < 0.0001 versus normal group. **P < 0.01; ***P < 0.001 versus STS group. (D) HepG2 cells were pretreated with indicated concentrations of PD98059 and challenged with or without 10 nm STS for additional 24 h. The expression levels of ERK and p‐ERK were detected by western blotting. β ‐tubulin was used as an internal control. (E) HepG2 cells were pretreated with indicated concentrations of Go6983 or NAC and incubated additional 24 h with or without 10 nm STS. The mRNA levels of ChREBPα and β were measured by quantitative RT‐PCR. Left panel shows the relative expression level of ChREBPα after normalization to β‐actin. The right panel shows the relative expression level of ChREBPβ normalized by ChREBPα. Data from one representative experiment of three independent experiments are presented as the mean ± SE of three technical replicates. One‐way ANOVA test: ###*P* < 0.001; ####*P* < 0.0001 versus normal group.

STS at lower concentrations is also an ERK activator [30, 31] although, at high concentrations, it is also a well‐known PKC inhibitor and apoptosis inducer. Thus, we next evaluated the effect of an STS treatment on ChREBP expression in a liver cell line. An initial dose response of STS was tested in HepG2 cells. Cultured cells were exposed to from 0 to 10 nm STS since no obvious effects on cell proliferation or survival were observed at least for 48 h (data not shown). The mRNA expression levels of ChREBPα and β at 24 h after the STS treatment were decreased in a dose‐dependent manner (Fig. 4A). When examined at a concentration of 10 nm, the expression levels of ChREBPα and β were decreased by the STS treatment in a time‐dependent manner (Fig. 4B). To explore the mechanism responsible for the inhibition of STS on ChREBP expression, the role of ERK signaling was investigated. ERK was phosphorylated within 15 min by a 10‐nm STS treatment (Fig. 5A) and the effect remained after a 24‐h incubation period (Fig. 5B). Furthermore, pretreatment with PD98059 abolished the effect of STS on the gene expression of ChREBPα and β (Fig. 5C). The efficacy of PD98059 was further confirmed by western blotting. As shown in Fig. 5D, the administration of STS resulted in the phosphorylation of ERK and the level of phosphorylated ERK was significantly reduced by a PD98059 pretreatment. This result indicates that PD98059 substantially suppressed the activation of the MEK‐ERK pathway caused by STS. However, pretreatments with Go6983 or NAC showed no effects on the STS elicited suppression of ChREBPα and β expression (Fig. 5E). Similar but different from the effect of H_2_O_2_, this implies that the STS triggered suppression of ChREBP expression also requires ERK activation, but that oxidative stress was not involved. A possible explanation for this is that a low concentration of STS is ineffective in generating oxidative stress in HepG2 cells. Furthermore, western blotting revealed that the protein expression level of L‐PK, the downstream target gene of ChREBP, was reduced by the STS treatment (Fig. S2A). Quantitative analysis of the mRNA expression levels of the enzymes also showed significantly lower expressions of L‐PK, fatty acid synthase (FAS), and acetyl‐CoA carboxylase (ACC; Fig. S2B), which are consistent with the data observed in ChREBP‐deficient mice [32]. These results suggest that glycolysis and lipogenesis are also suppressed following ERK activation.

Taken together, we conclude that as long as ERK is activated by ROS, TPA, STS, etc., the expression of both ChREBPα and β is reduced, which indicates that the ERK pathway plays a prominent role in the transcriptional regulation of both isoforms, although the mRNA levels of ChREBPβ are < 1% that of ChREBPα in HepG2 cells. Namely, the gene expression of ChREBPα is very sensitive to ERK signaling in liver cells. This investigation expands our current understanding of the response of ChREBPα and β to effectors other than carbohydrates and highlights a more potent role of hepatic ChREBPα rather than β in response to oxidative stress. As a result, the decrease in ChREBPα indicates that a reduced level of hepatic fatty acid synthesis and glycolysis may play a protective role in response to oxidative stress. Paradoxically, Sirek et al. reported that an insulin treatment increased the expression of ChREBPα through the Pak1/MEK/ERK/Oct‐1 signaling cascade in mouse hepatocytes [33, 34]. The insulin treatment promoted the expression of ChREBPα through ERK activation, a process that involved the inactivation of oct‐1, which, contrary to our findings, indicates that the activation of ERK suppresses ChREBPα and β expression. However, it is well known that ChREBP is primarily controlled via its cytosol‐nuclei shuttle by elevated glucose levels, while insulin was a potent inducer of sterol regulatory element‐binding proteins (SREBP)‐1c [35]. The issue of whether insulin can activate the expression of ChREBP independent of glucose stimuli remains controversial. We also attempted to stimulate HepG2 cells with insulin, but no effect on the expression of ChREBP was detected (data not shown). Moreover, glucose activates the hepatic gene expression of ChREBP independent of insulin signaling although it can be activated by additional carbohydrates [36, 37]. On the other hand, fructose administered by gavage robustly increased ChREBPβ expression but not ChREBPα in the intestines of mice [38]. For the above reasons, specific isoforms of ChREBP should be studied separately when referring to any exogenous stimulus in different tissues. Further study will also be required to elucidate the mechanism of ChREBP expression at the transcriptional level including the involvement responsible for representative signaling pathways such as JNK and p38 induced by oxidative stress as well as ERK signaling.

## Conflict of interest

The authors declare no conflict of interest.

## Author contributions

LL conceived and wrote the manuscript. LL, HS, and HE designed and carried out the experiments. NF and DY performed the statistical analyses. HS and KS analyzed the data. NF and HS assisted in writing the manuscript. All of the authors have read and approved the final version of the manuscript.

## Supporting information

Fig. S1. Analysis of agarose gel electrophoresis of the ChREBPα and β mRNA expression by H_2_O_2_ treatment in HepG2 cells.
Fig. S2. Proteins and genes involved in glycolysis and lipogenesis by STS treatment in HepG2 cells.

## Data Availability

The analyzed data sets generated during the study are available from the corresponding author on reasonable request.
