# Supplementary material for: Activation of the mitogen‐activated protein kinase ERK1/2 signaling pathway suppresses the expression of ChREBPα and β in HepG2 cells
Source: FEBS Open Bio. 2021 Jun 17;11(7):2008–18. doi: 10.1002/2211-5463.13208 (PMC8255832; doi:10.1002/2211-5463.13208)
Supplement: Supplementary file 1 — Fig. S1. Analysis of agarose gel electrophoresis of the ChREBPα and β mRNA expression by H2O2 treatment in HepG2 cells. Fig. S2. Proteins and genes involved in glycolysis and lipogenesis by STS treatment in HepG2 cells. [file FEB4-11-2008-s001.docx]

**
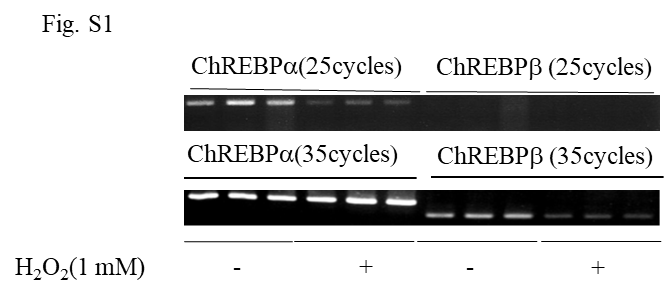
**

**Fig. S1 Analysis of** **agarose gel electrophoresis of the ChREBPα and β mRNA expression by H_2_O_2_ treatment in HepG2 cells.**

HepG2 cells were treated with or without 1 mM H_2_O_2_ for 24 hours. The mRNA levels of ChREBPα and β were quantified by agarose gel electrophoresis. The upper panel shows the mRNA levels of ChREBPα and β for 25 PCR cycles. The lower panel shows the mRNA levels of ChREBPα and β for 35 PCR cycles.


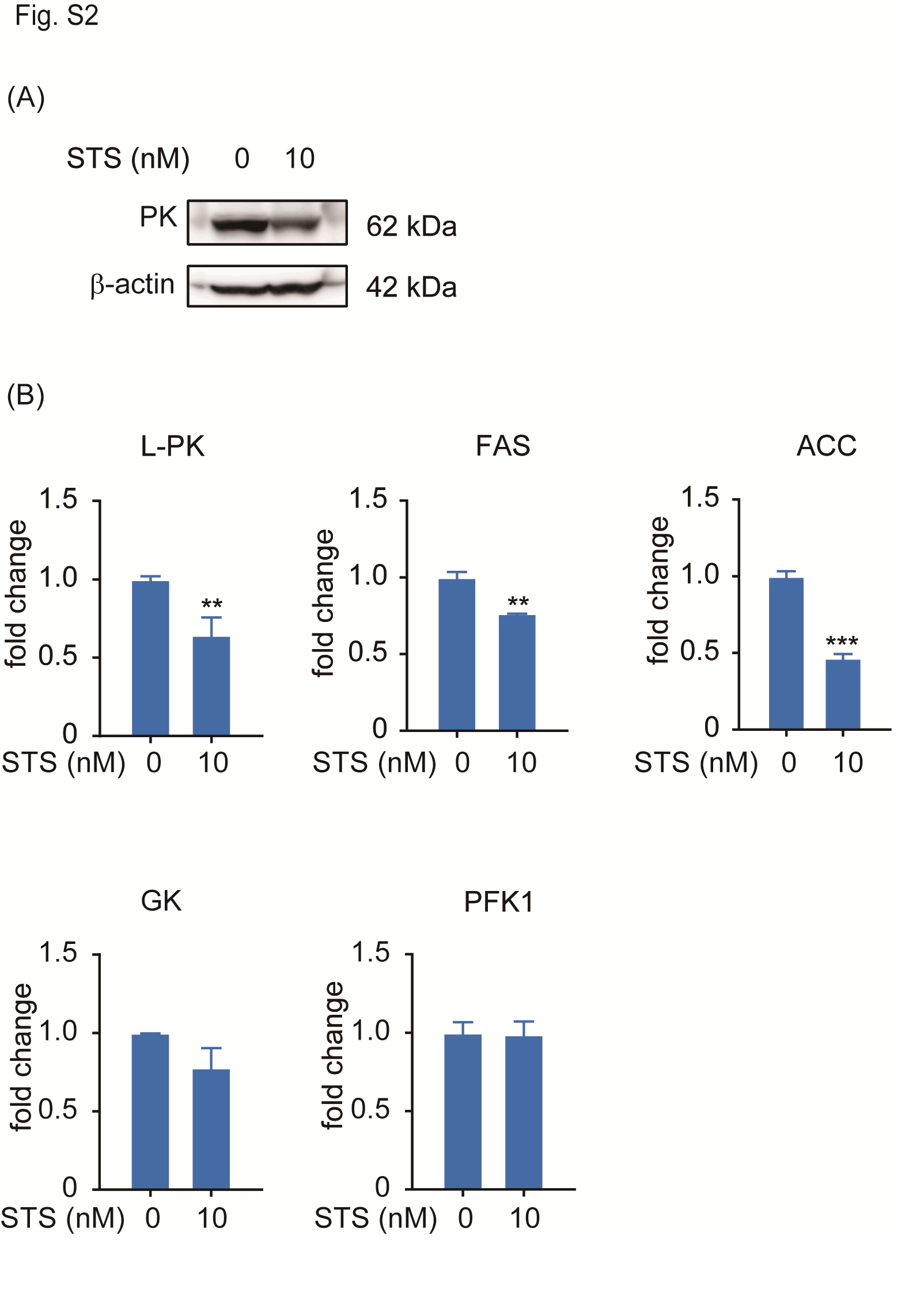


**Fig. S2 Proteins and genes involved in glycolysis and lipogenesis by STS treatment in HepG2 cells.**

(A) HepG2 cells were treated with 10 nM STS for 24 hours. At the end of incubation, the protein expression level of L-PK was determined by western blotting. The expression of β-actin was used as a loading control.

(B) HepG2 cells were challenged with 10 nM STS for 24 hours. The mRNA levels of L-PK, FAS, ACC, GK and PFK1, were measured by q-RT-PCR. Data from one representative experiment of three independent experiments is presented as the mean ± S.E. of three technical replicates. Student t test: **, p < 0.01; ***, p < 0.001 versus normal group.
